# Retraction of: Lipidomics, transcription analysis, and hormone profiling unveil the role of *CsLOX6* in MeJA biosynthesis during black tea processing

**DOI:** 10.1093/hr/uhae146

**Published:** 2024-06-03

This is a retraction of: Gaoyang Zhang, Jingjing Wei, Linyan Li, Dandan Cui, Lipidomics, transcription analysis, and hormone profiling unveil the role of *CsLOX6* in MeJA biosynthesis during black tea processing, *Horticulture Research*, Volume 11, Issue 3, March 2024, uhae032, https://doi.org/10.1093/hr/uhae032

In April 2024, the journal was alerted to concerns about the above-mentioned article, including allegations regarding both the inclusion of data without appropriate authorizations and correct authorship attributions. The journal subsequently investigated, and the corresponding author has acknowledged the lack of necessary permissions for some of the data used and has apologized for the inconvenience caused. For these reasons, the corresponding author and Editors have decided to retract this article.

